# On-Demand Droplet Routing and Splitting Using Independently Addressable Interdigitated Electrodes

**DOI:** 10.3390/mi17030375

**Published:** 2026-03-20

**Authors:** Yunus Aslan

**Affiliations:** Department of Electrical and Electronics Engineering, Middle East Technical University, Ankara 06800, Turkey; aslany@metu.edu.tr

**Keywords:** microfluidics, droplet splitting, droplet sorting, dielectrophoresis

## Abstract

Droplet microfluidics enables precise manipulation of picoliter-to-nanoliter-scale droplets and supports key operations such as merging, splitting, sorting, and trapping, facilitating controlled handling of minute fluid volumes. These capabilities have significantly advanced high-throughput drug discovery, single-cell analysis, molecular diagnostics, and synthetic biology. Among these operations, droplet splitting is particularly important for multi-step biochemical assays and parallel processing. Splitting strategies can be broadly categorized as passive, relying on channel geometry or microstructures, or active, employing external stimuli such as thermal, magnetic, acoustic, or electric fields. Electric-field-based methods are especially attractive due to their rapid response and tunability; however, many reported systems require relatively high operating voltages. Here, we present a low-voltage microfluidic platform that integrates tilted interdigitated electrodes (IDEs) with an asymmetric Y-junction to enable electrically tunable droplet splitting and sorting within a single device architecture. Two independently addressable tilted IDE arrays generate localized electric-field gradients that induce dielectrophoretic droplet deflection at moderate voltages. By adjusting the applied voltage amplitude and selectively activating the electrode arrays, droplets can be dynamically routed into designated outlets or deterministically split in real time, providing adaptable electrohydrodynamic control with minimal structural complexity.

## 1. Introduction

Droplet manipulations encompass the precise control of the formation, transport, and interaction of individual fluid droplets within microfluidic environments, typically at volumes from picoliters to nanoliters [1,2]. This capability enables the implementation of various microfluidic operations such as merging [3,4], splitting [5,6], sorting [7,8], and trapping [9]. The precise control of such minute volumes has enabled transformative applications across a broad range of fields, including particle synthesis [10], drug discovery [11], single-cell analysis [12,13], molecular diagnostics [14,15], and directed evolution [16].

Droplet splitting, the controlled division of a parent droplet into multiple daughter droplets, is a key operation that facilitates accurate droplet partitioning and directional routing, which are essential for high-throughput analysis, multi-step biochemical assays, and parallel sample processing. Splitting strategies are broadly categorized as passive or active. Passive methods rely on channel geometry and embedded microstructures, such as obstructions [17], T-junctions [18], Y-junctions [19,20], and multi-furcating channels [21], where intrinsic hydrodynamic forces govern droplet deformation and division. While these approaches are simple to fabricate and operate, they are inherently static and lack real-time adaptability. Active methods, in contrast, employ external energy fields, including valves [22], magnetic [23], electric [24,25], and acoustic [26,27], to dynamically control droplet splitting. Active techniques offer superior operational flexibility, on-demand tunability, and real-time control over droplet behavior, enabling more versatile microfluidic workflows.

Electrical methods are particularly attractive among active droplet manipulation strategies due to their straightforward integration with microfluidic architectures, rapid response times, and precise tunability through simple adjustments of voltage or frequency [28]. These methods rely on electromechanical forces induced by an applied electric field, commonly referred to as electrohydrodynamic (EHD) forces, which can be classified into electrowetting on dielectric (EWOD) and dielectrophoresis (DEP) [29]. In EWOD, patterned electrodes dynamically modulate surface wettability to control droplet motion, whereas DEP arises from forces exerted on electrically neutral particles in a non-uniform electric field. Implementing DEP in microfluidic systems requires electrodes capable of generating spatially non-uniform electric fields. Conventional approaches often use three-dimensional electrodes formed by filling microchannels with conductive fluids such as saltwater or liquid metal [30]. While effective, these electrodes typically produce poorly localized fields and require high operating voltages (e.g., kV). By contrast, interdigitated electrodes (IDEs) patterned on the channel floor generate highly localized electric fields, enabling precise droplet manipulation at much lower voltages, and have been successfully applied for both sorting [31] and merging [32] operations. Owing to its robustness and tunability, the proposed platform establishes a compact and electrically controllable electrohydrodynamic framework, enabling precise cell handling, droplet-based biochemical assays, and intracellular delivery strategies [33,34,35,36], and thereby providing a versatile foundation for next-generation lab-on-a-chip technologies in drug screening, molecular diagnostics, particle synthesis, single-cell processing, and synthetic biology.

**Figure 1:**
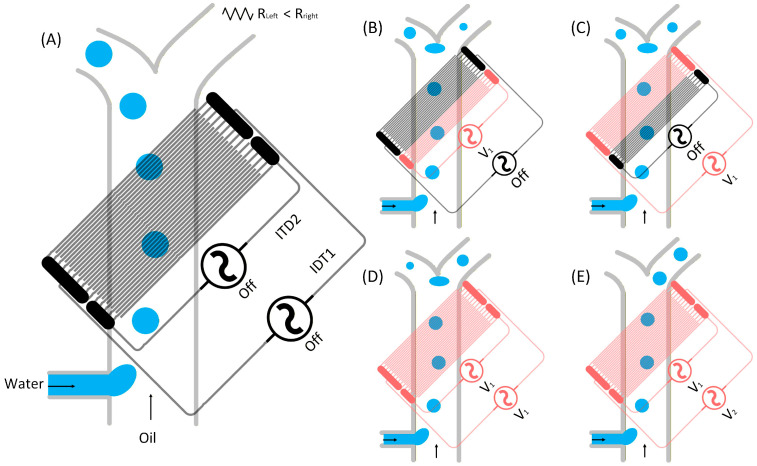
Schematic of the microfluidic device integrating a T-junction for droplet generation and an asymmetric Y-junction (right arm approximately three times longer than the left). Two tilted IDE arrays (20-finger and 10-finger pairs, angled at ~45° and positioned ~20 µm beneath the microchannel) are patterned beneath the junction region and can be actuated independently. Different operating modes can be implemented (IDE1 on, IDE2 on, or IDE1 + IDE2 on) by applying independent AC voltages (V_1_ and V_2_) to manipulate droplet trajectories. (**A**) In the absence of an applied voltage, droplets preferentially enter the left outlet due to the hydrodynamic resistance mismatch. (**B**) When IDE2 is activated above a threshold voltage, droplets are deflected toward the right branch. (**C**) Activating IDE1 alone at the same voltage produces a larger deflection owing to its higher number of electrodes. (**D**) Simultaneous activation of both IDE arrays further increases the lateral displacement. (**E**) Adjusting the applied voltages (V_1_ and V_2_) above the sorting threshold enhances droplet deflection, and at sufficiently high voltages, complete sorting into the right outlet is achieved.

Leveraging the localized control provided by IDEs, a low-voltage electric field-based microfluidic platform is presented for precise droplet splitting, which can also be readily adapted for droplet sorting. The key concept is to exploit DEP-induced droplet deflection as a deterministic and tunable mechanism to trigger droplet splitting at an asymmetric Y-junction, enabling on-demand control beyond passive geometric breakup. Tilted IDE arrays are integrated beneath the asymmetric Y-junction to generate localized electric-field gradients and achieve controlled droplet routing at only a few volts. Two independently addressable IDE arrays (20 and 10 fingers) enable tunable deflection through selective activation and voltage adjustment, while manipulation performance can be further optimized by controlling the number of activated electrodes. Increasing the number of activated electrodes broadens the effective field region and enhances DEP-induced deflection efficiency. As illustrated in Figure 1, droplets generated at a T-junction enter the asymmetric Y-junction and preferentially flow into the shorter outlet in the absence of actuation. Upon AC excitation, droplets are progressively deflected toward the longer outlet, with increased displacement obtained at higher voltages or with simultaneous activation of both arrays, ultimately enabling complete switching of droplets to the opposite branch.

## 2. Materials and Methods

### 2.1. Chip Fabrication

The microfluidic device was fabricated through a multi-step process. Glass coverslips were thoroughly cleaned using sequential washes in acetone, methanol, and isopropyl alcohol (10 min each), followed by nitrogen drying and thermal dehydration at 120 °C for 15 min. Microchannel patterns were created using standard photolithography techniques: SU-8 3550 photoresist was spin coated onto the cleaned substrates at 3000 rpm for 30 s, soft baked at 95 °C for 20 min, and exposed to UV light (365 nm, 50–75 s) through an acetate mask. After post exposure baking for 5 min, the patterns were developed using developer (4 min immersion). PDMS (Sylgard 184, Dow Corning, Midland, MI, USA) prepolymer was mixed at a 10:1 base to curing agent ratio, degassed, and cured on the masters at 80 °C for 2 h. The cured PDMS was then bonded to the patterned substrates using oxygen plasma treatment (90 W, 30 s), and fluid access ports (1 mm diameter) were created using biopsy punches.

### 2.2. Electrode Fabrication

Electrodes were fabricated on glass substrates. A ≈ 2 µm thick layer of S1818 photoresist was spin coated at 4000 rpm for 30 s and soft baked at 90 °C for 4 min. The substrates were then exposed to UV light, and the exposed patterns were developed for 2 min, yielding well-defined electrode structures. A bilayer metal deposition was performed, consisting of a 10 nm titanium adhesion layer followed by 200 nm of gold. Excess metal was removed via a lift-off process by immersing the substrates in acetone for 20 min in sealed containers, yielding clean and well-defined electrodes.

### 2.3. Experimental Setup and Characterization

Single emulsion droplets were produced using mineral oil (Product No: M5904, Sigma-Aldrich, St. Louis, MO, USA) as the continuous phase, supplemented with 1% *w*/*v* Span 80 (Product No: S6760, Sigma-Aldrich) as the surfactant. Electrode actuation and droplet motion control were achieved using function generators (Keysight, Santa Rosa, CA, USA). A 10 kHz sinusoidal signal with varying amplitudes was applied to designated electrode sets on the microfluidic device to generate the electric field for droplet control. Droplet videos were acquired using a microscope imaging system (Eclipse Ci-L, Nikon, Tokyo, Japan). The recorded videos were analyzed using Fiji software (Version 1.54k, Bethesda, MD, USA) [37], and representative frames were extracted to generate the figures.

### 2.4. Theoretical Background

The droplet routing and splitting behavior in the present device arises from the interplay between hydrodynamic resistance asymmetry and electrically induced DEP forcing. In the absence of electric actuation, droplet trajectories are governed by pressure-driven flow within the asymmetric Y-junction. The hydrodynamic resistance of a rectangular microchannel under fully developed laminar flow can be expressed as [38,39]:

(1) $$R=\frac{12\mu L}{h^{3}w}{\left[1-\sum_{n,odd}^{\infty }\frac{1}{n^{5}}\times \frac{192}{\pi^{5}}\times \frac{h}{w}tanh(\frac{n\pi w}{2h})\right]}^{-1},$$

where *μ* is the dynamic viscosity of the continuous phase, *L* is the channel length, *w* is the channel width, and *h* is the channel height. Since both outlet branches possess identical cross-sectional dimensions, the hydraulic resistance scales linearly with channel length (*R ∝ L*). Consequently, the longer outlet branch exhibits higher resistance, leading to a pressure-driven flow bias toward the shorter outlet under field-free conditions.

Upon activation of the IDEs, a spatially non-uniform electric field is established in the channel, giving rise to dielectrophoretic forcing on the droplet. The time-averaged DEP force acting on a spherical droplet of radius r suspended in a medium with permittivity *εₘ* under AC excitation is given by:

(2) FDEP=2πr3εmRe[K(ω)]∇∣E∣2,

where K(ω) is the Clausius–Mossotti factor determined by the complex permittivities of the droplet and the surrounding medium, and $\nabla |E{|}^{2}$ denotes the gradient of the squared electric-field magnitude. Under positive DEP conditions, droplets are attracted downward toward the electrode surface. Rather than being trapped at the initial point of contact, the tilted electrodes function as electrohydrodynamic ‘rails’. The droplets are guided to remain within the high-field intensity zones while being simultaneously transported upward by the hydrodynamic drag. This dynamic interaction forces the droplets to slide laterally along the oblique longitudinal axis of the electrodes.

Under low Reynolds number conditions, the hydrodynamic drag force can be approximated by Stokes’ law:

(3) $$F_{DRAG}=6\pi \mu rv$$

where *v* is the droplet velocity. While hydrodynamic drag governs longitudinal transport along the channel, redirection occurs when the pDEP-induced guiding effect constrains the droplets to slide laterally along the tilted electrode axes. This lateral displacement allows the droplets to overcome the pressure-driven hydrodynamic bias toward the lower-resistance branch of the asymmetric junction. By adjusting the applied voltage and flow rate, the balance between dielectrophoretic guiding and hydrodynamic transport can be modulated, enabling controlled droplet redirection and splitting.

## 3. Results

The microfluidic device integrated three functional modules: droplet generation, droplet splitting, and an optional droplet sorting unit. Droplets were produced using a T-junction geometry, selected for its simplicity, robustness, and widespread adoption [40]. In this configuration, the continuous oil phase sheared the dispersed aqueous phase at the junction, generating discrete aqueous droplets suspended within the immiscible oil phase. For active droplet manipulation, two IDE array electrodes were embedded beneath the main channel at an angle of ≈45° relative to the flow direction. This orientation introduces a transverse electric-field component while maintaining an extended interaction region for lateral droplet deflection. The two IDE arrays consisted of 20 and 10 fingers, respectively, providing extended spatial coverage of the active region during droplet transit and generating a non-uniform electric-field distribution within the electrode area. The electrode-to-channel spacing was set to 20 µm to establish a strong electric-field gradient within the fluidic domain. Downstream, the channel terminated in an asymmetric Y-bifurcation located approximately 1.25 mm from the last tilted electrode. The left branch (5 mm) had approximately one-third the length of the right branch, introducing a hydrodynamic resistance imbalance that biased droplets toward the shorter branch under field-free conditions.

Upon application of an AC voltage, droplets traversing the electrode region experienced a lateral positive dielectrophoretic (pDEP) force superimposed on the forward hydrodynamic drag. Owing to their strong dielectric contrast and higher conductivity relative to the surrounding oil [41], droplets responded effectively to the applied field. Importantly, under the operating conditions used here, the direction of the pDEP force remained largely frequency-independent. The combined action of hydrodynamic drag and pDEP enabled precise diversion of droplets from their native streamline, facilitating controlled routing toward alternative outlets. By tuning the amplitude of the applied voltage and selectively energizing either IDE array 1, IDE array 2, or both, the degree of lateral deflection at the Y-junction could be finely regulated, allowing the system to operate interchangeably as a droplet splitter or a droplet sorter.

**Figure 2:**
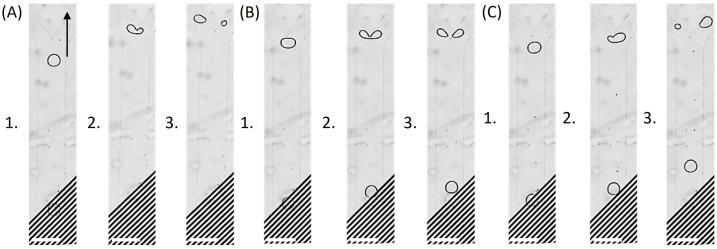
Voltage-controlled droplet deflection and splitting at the Y-junction with IDE1 electrodes energized. (**A**) At low voltage (3 Vp), droplets exit predominantly into the left channel. (**B**) At intermediate voltage (5 Vp), ~50% of the droplet volume is redirected to the right channel. (**C**) At higher voltage (7 Vp), most droplets flow into the right channel. Flow rates: 1 µL/min (side junctions) and 5 µL/min (main channel). Arrow indicates flow direction. Subpanels (1–3) show the droplet approaching the junction (1), at the junction (2), and after passing the junction (3). Scale bar: 250 µm.

For instance, in the absence of an applied electric field, all droplets exited through the left outlet of the asymmetric Y-junction at flow rates of 1 µL/min for each side inlet and 5 µL/min for the main channel. Upon energizing the first IDE array, the droplet distribution between the left and right outlets became strongly dependent on the applied voltage. Below a threshold amplitude (≤2 Vp), no appreciable deflection was observed. At intermediate voltages (3–5 Vp), an increasing fraction of the droplet volume was redirected into the right outlet: 14.4% at 3 Vp (Figure 2A), 31.6% at 4 Vp, and nearly equal partitioning (50.2%) at 5 Vp (Figure 2B). At higher voltages, droplet redistribution strongly favored the right outlet, with 87.9% of the droplet volume directed there at 7 Vp (Figure 2C).

**Figure 3:**
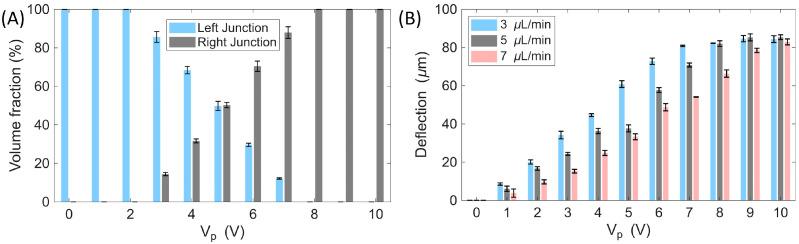
Voltage- and flow-rate-dependent droplet volume distribution and lateral deflection. (**A**) Droplet volume fractions at the left and right outlets as a function of applied voltage at a main-channel flow rate of 5 µL/min. (**B**) Lateral droplet deflection as a function of applied voltage and flow rate (3, 5, and 7 µL/min), showing enhanced deflection at higher voltages and reduced deflection at higher flow rates. Error bars represent measurements from three consecutive droplets (*n* = 3).

A quantitative summary of the droplet volume fractions at the Y-junction and the corresponding lateral deflection as a function of the applied voltage is presented in Figure 3A. Additional analysis performed at main-channel flow rates of 3 and 7 µL/min confirmed the same overall trend (Figure 3B), with increasing voltage progressively redirecting a larger fraction of droplets into the right branch. Consistent with these observations, the lateral droplet displacement exhibited a monotonic increase with voltage, while also showing a clear dependence on flow rate. At 10 Vp, maximum lateral displacements of approximately 84.3 µm, 85.3 µm, and 82.9 µm were obtained at flow rates of 3, 5, and 7 µL/min, respectively. At low voltages (1–2 Vp), deflection remained minimal (≈8.5–20.0 µm at 3 µL/min; 6.0–16.6 µm at 5 µL/min; 3.8–9.6 µm at 7 µL/min). At intermediate voltages (3–6 Vp), the lateral displacement progressively intensified, before approaching saturation at higher voltages (7–10 Vp), where further increases resulted in only minor changes.

**Figure 4:**
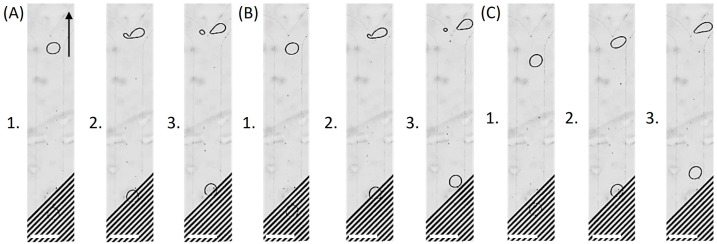
IDE-controlled droplet splitting and sorting at the asymmetric Y-junction. (**A**) Activating IDE2 at 0.5 Vp in addition to IDE1 generated a stronger and more spatially extended electric field, enhancing droplet deflection toward the right outlet. (**B**) Increasing the IDE2 amplitude to 1 Vp while keeping both IDE arrays active further strengthened the field and redirected a larger fraction of the droplet volume into the right branch. (**C**) Further increasing IDE2 to 2 Vp redirected all droplets into the right outlet, demonstrating complete control over droplet routing. Subpanels (1–3) show the droplet approaching the junction (1), at the junction (2), and after passing the junction (3). Scale bar: 250 µm.

Next, the effect of the number of activated electrodes on droplet trajectories was investigated by selectively energizing the two IDE arrays. Compared with IDE1-only operation (Figure 2C), simultaneous activation of both arrays (IDE1: 7 Vp, IDE2: 0.5 Vp) generated a stronger and more spatially extended electric field, which enhanced the DEP-driven deflection force and directed a larger fraction of the droplet volume into the right branch (Figure 4A). As the IDE2 amplitude was further raised, the lateral displacement became progressively more pronounced, ultimately enabling complete droplet routing into the right outlet and demonstrating full sorting capability (Figure 4B,C).

**Figure 5:**
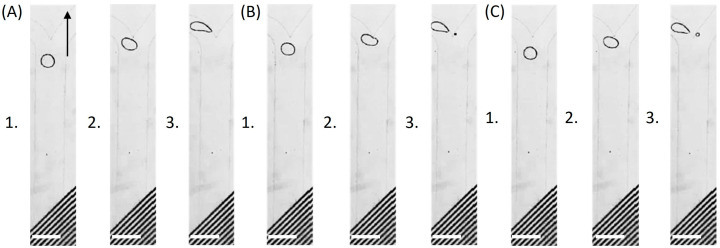
Voltage-controlled droplet routing with selective activation of IDE2. (**A**) Under field-free conditions, droplets exit exclusively through the left outlet due to the inherent hydrodynamic resistance asymmetry of the Y-junction. (**B**) When IDE2 is activated at 4 Vp, a lateral DEP force is induced, leading to slight droplet partitioning toward the right branch. (**C**) Increasing the applied voltage to 5 Vp further amplifies the electric-field gradient within the junction, resulting in pronounced lateral deflection toward the right outlet. Subpanels (1–3) show the droplet approaching the junction (1), at the junction (2), and after passing the junction (3). Scale bar: 250 µm.

To further elucidate the electrohydrodynamic balance governing this transition, an amplitude sweep of the IDE2 activation voltage was also performed. In the absence of electric actuation (Figure 5A), droplets exited exclusively through the left outlet due to the intrinsic hydrodynamic resistance asymmetry of the Y-junction. Activation of IDE2 at 4 Vp (Figure 5B) generated a lateral DEP force, resulting in a slight volumetric transfer toward the right branch. Increasing the amplitude to 5 Vp (Figure 5C) strengthened the electric-field gradient, thereby enhancing the transverse DEP forcing and producing pronounced lateral deflection toward the right outlet, accompanied by increased volumetric partitioning into the right branch.

**Figure 6:**
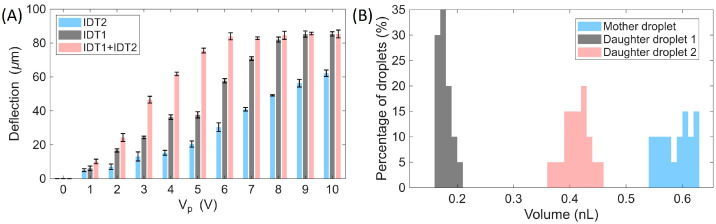
Effect of electrode activation on droplet deflection and splitting performance. (**A**) Droplet deflection as a function of driving amplitude (Vp) under three actuation conditions: IDE1 only, IDE2 only, and both IDE arrays activated. Error bars represent measurements from three consecutive droplets (*n* = 3). (**B**) Monodispersity analysis of droplet splitting (IDE1 = 4 Vp, IDE2 off). The volume fractions of the mother droplet and the two daughter droplets (left branch: Daughter 1; right branch: Daughter 2) were measured over 20 consecutive splitting events, yielding CVs of 4.60%, 5.82%, and 4.98%, respectively, confirming monodisperse splitting.

Overall, the droplet trajectory analysis confirmed that the total deflection increased with both the number of energized electrodes and the applied voltage. Quantitative comparisons for the cases where only IDE1 was activated, only IDE2 was activated, and both IDE arrays were activated are summarized in Figure 6A. These results demonstrate that droplet routing can be finely tuned by adjusting the number of activated electrodes and the driving amplitude, even at relatively low voltages, highlighting the efficiency of the proposed electrode design for low-power droplet manipulation. In addition, the monodispersity of droplet splitting was assessed by measuring the volume fractions of the mother droplets prior to splitting and the two daughter droplets after splitting over 20 consecutive events (Figure 6B). The coefficient of variation (CV = standard deviation/mean) [37] was calculated for each population, yielding CVs of 4.60% for the mother droplets, 5.82% for Daughter 1 (left branch), and 4.98% for Daughter 2 (right branch), confirming monodisperse splitting performance.

Finally, the influence of hydrodynamic variations on droplet behavior was examined by adjusting the main-channel flow rate (Figure S1). Increasing the flow rate produced smaller droplets and reduced their residence time within the electrode region. Under these conditions, splitting was not observed at high flow rates despite voltage actuation across the tested range (0–10 Vp), indicating that sufficient interaction time and droplet deformation are required for effective routing. At low flow rates, larger droplets occupied a substantial fraction of the channel cross-section, leading to strong geometric confinement within the junction. This confinement limited the ability of lateral DEP forcing to fully redirect the droplet, even at elevated voltage amplitudes, preventing complete sorting. In contrast, intermediate flow rates provided a balance between droplet size, confinement, and interaction time, enabling effective lateral deflection and deterministic routing. Overall, these observations show that routing performance depends on the interplay between electric forcing and hydrodynamic conditions, rather than on voltage magnitude alone.

Taken together, these experimental findings demonstrate that controllable droplet routing in the present system arises from the interplay between intrinsic hydrodynamic resistance asymmetry and spatially distributed DEP forcing. Conventional passive droplet splitting methods typically rely on symmetric or asymmetric channel geometries and carefully tuned flow-rate ratios to achieve volume partitioning. In such systems, splitting behavior is largely predetermined by channel design and offers limited dynamic control after fabrication. Active droplet cutting strategies reported in the literature include acoustic, thermal, electrowetting, and high-voltage DEP-based approaches. While these methods enable external actuation, many require complex device architectures, localized heating, mechanical components, or relatively high operating voltages. In contrast, the present approach integrates planar tilted IDE arrays beneath a fixed asymmetric junction, enabling electrically tunable droplet splitting at moderate voltages (≤10 Vp) without modifying channel geometry or flow-rate ratios. Furthermore, we demonstrate that both the applied voltage amplitude and the number of activated electrode fingers influence droplet routing behavior, providing an additional degree of electrical control over the partitioning dynamics. This electrohydrodynamic coupling between intrinsic hydrodynamic bias and adjustable DEP forcing constitutes a distinct operational strategy, yielding a compact, reversible, and electrically reconfigurable platform for controlled droplet cutting and routing within a single microfluidic architecture.

## 4. Conclusions

A low-voltage microfluidic device was developed for precise droplet manipulation by integrating tilted interdigitated electrodes with a Y-junction geometry. By combining tilted interdigitated electrodes with an asymmetric Y-junction, the system delivers precise, real-time control over droplet splitting and sorting at low operating voltages. Importantly, DEP-driven droplet deflection enables on-demand positioning of droplets at the junction, allowing controlled and tunable splitting without modifying the device architecture or flow conditions. The scalable electrode architecture enables stronger and more spatially distributed electric-field gradients, and the manipulation performance can be further tuned by selectively activating different numbers of electrodes, improving deflection efficiency at low voltages. Owing to its robustness and tunability, the platform provides a promising foundation for next-generation microfluidic technologies in drug screening, particle synthesis, single-cell processing, molecular diagnostics, and synthetic biology.

## Data Availability

The data supporting the findings of this study are provided as Supplementary Material.

## Supplementary Materials

The following supporting information can be downloaded at: https://www.mdpi.com/article/10.3390/mi17030375/s1: Figure S1. Influence of droplet size on voltage-controlled droplet routing; Dataset (Excel file) containing numerical data for the figures in the manuscript.

## Abbreviations

The following abbreviations are used in this manuscript:

AC	Alternating current
CV	Coefficient of variation
DEP	Dielectrophoresis
EHD	Electrohydrodynamic
EWOD	Electrowetting-on-dielectric
IDE	Interdigitated electrode
pDEP	Positive dielectrophoresis
PDMS	Polydimethylsiloxane
UV	Ultraviolet
