# Supplementary material for: On-Demand Droplet Routing and Splitting Using Independently Addressable Interdigitated Electrodes
Source: Micromachines (Basel). 2026 Mar 20;17(3):375. doi: 10.3390/mi17030375 (PMC13028701; doi:10.3390/mi17030375)
Supplement: Supplementary file 1 [file micromachines-17-00375-s001.zip › Supplementary_Information.pdf]

On-Demand Droplet Routing and Splitting Using Independently Addressable Interdigitated Electrodes

Yunus Aslan

Department of Electrical and Electronics Engineering, Middle East Technical University, Ankara 06800, Turkey; aslany@metu.edu.tr

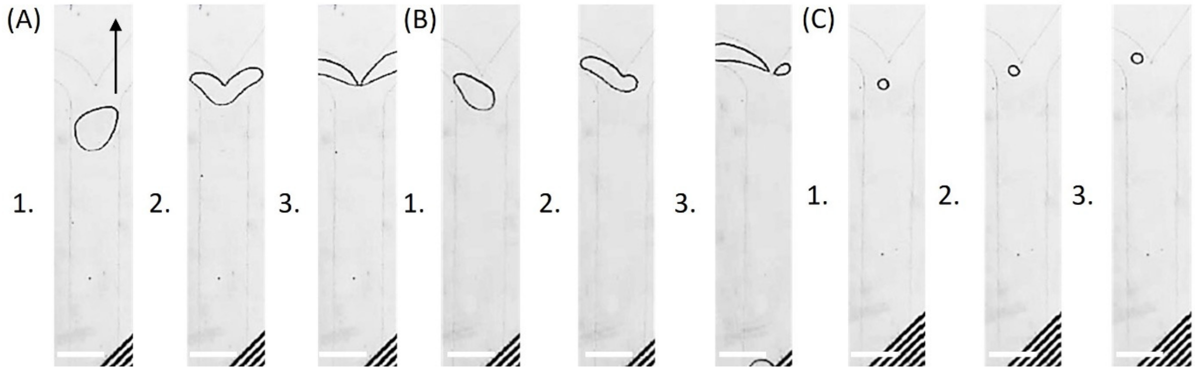

Figure S1. Influence of droplet size (flow-rate-dependent) on voltage-controlled droplet routing. (A) At low flow rates (main channel: 0.5 μL/min), larger droplets experience strong geometric confinement within the junction, which limits complete redirection toward the right outlet even at elevated voltages (7 Vp). (B) When the electrodes are deactivated (main channel: 1 μL/min), droplets follow the intrinsic hydrodynamic bias and exit through the left outlet; under these conditions, complete redirection is not possible, and droplet splitting occurs at the junction. (C) At high flow rates (main channel: 12 μL/min), smaller droplets and reduced residence time within the electrode region suppress effective lateral deflection despite voltage actuation (10 Vp), thereby preventing splitting. All subpanels (1–3) within each condition show the same droplet at different time points as it traverses the junction.
